# Improving the Yield of Genetic Diagnosis through Additional Genetic Panel Testing in Hereditary Ophthalmic Diseases

**DOI:** 10.3390/cimb46050300

**Published:** 2024-05-20

**Authors:** Jin Gwack, Namsu Kim, Joonhong Park

**Affiliations:** 1 Department of Preventive Medicine, Jeonbuk National University Medical School, Jeonju 54907, Republic of Korea; gwackjin@jbnu.ac.kr; 2 Department of Laboratory Medicine, Jeonbuk National University Medical School and Hospital, Jeonju 54907, Republic of Korea; namsu1224@naver.com; 3 Research Institute of Clinical Medicine of Jeonbuk National University-Biomedical Research Institute of Jeonbuk National University Hospital, Jeonju 54907, Republic of Korea; 4 Department of Laboratory Medicine, Daejeon St. Mary’s Hospital, Daejeon 34943, Republic of Korea

**Keywords:** massively parallel sequencing, gene panel sequencing, genetic diagnosis, hereditary ophthalmic diseases, precision medicine

## Abstract

Numerous hereditary ophthalmic diseases display significant genetic diversity. Consequently, the utilization of gene panel sequencing allows a greater number of patients to receive a genetic diagnosis for their clinical manifestations. We investigated how to improve the yield of genetic diagnosis through additional gene panel sequencing in hereditary ophthalmic diseases. A gene panel sequencing consisting of a customized hereditary retinopathy panel or hereditary retinitis pigmentosa (RP) panel was prescribed and referred to a CAP-accredited clinical laboratory. If no significant mutations associated with hereditary retinopathy and RP were detected in either panel, additional gene panel sequencing was requested for research use, utilizing the remaining panel. After additional gene panel sequencing, a total of 16 heterozygous or homozygous variants were identified in 15 different genes associated with hereditary ophthalmic diseases. Of 15 patients carrying any candidate variants, the clinical symptoms could be tentatively accounted for by genetic mutations in seven patients. However, in the remaining eight patients, given the in silico mutation predictive analysis, variant allele frequency in gnomAD, inheritance pattern, and genotype–phenotype correlation, fully elucidating the clinical manifestations with the identified rare variant was challenging. Our study highlights the utility of gene panel sequencing in achieving accurate diagnoses for hereditary ophthalmic diseases and enhancing the diagnostic yield through additional gene panel sequencing. Thus, gene panel sequencing can serve as a primary tool for the genetic diagnosis of hereditary ophthalmic diseases, even in cases where a single genetic cause is suspected. With a deeper comprehension of the genetic mechanisms underlying these diseases, it becomes feasible.

## 1. Introduction

Hereditary ophthalmic diseases are genetically and clinically heterogeneous, affecting approximately 1 in 1000 people worldwide. These conditions encompass non-syndromic, syndromic, non-progressive, and progressive molecular pathologies, including hereditary optic neuropathies, retinal and corneal dystrophies, and other progressive ophthalmic diseases [1]. Progressive ophthalmic diseases leading to severe blindness or visual impairment affect 4 in 10,000 children each year [2]. Congenital glaucoma affects 1 in 20,000 children, and approximately 3 in 10,000 children under 15 years old are affected by congenital cataracts [3]. Albinism has a global prevalence of 1 in 20,000 [4]. Coloboma, anophthalmia, and microphthalmia occur in an estimated 1.19 per 10,000 children by age 16 [5]. Retinal dystrophies include rod-dominant diseases such as Leber congenital amaurosis, retinitis pigmentosa (RP), early-onset retinal dystrophy, and rod-cone dystrophy, as well as cone-dominant diseases like Stargardt disease, macular dystrophies, and cone/cone-rod dystrophy, with or without extraocular features [6]. Retinal dystrophies impact 2.2 in 10,000 children by age 16, with RP being the most common form [7]. In the Republic of Korea, the prevalence of visual impairment, myopia, hyperopia, and astigmatism in individuals over 5 years of age was 0.4 ± 0.1%, 53.7 ± 0.6%, 10.7 ± 0.4%, and 58.0 ± 0.6%, respectively. For participants over 3 years of age, the prevalence of strabismus and blepharoptosis was 1.5 ± 0.1% and 11.0 ± 0.8%, respectively. Among those over 40 years of age, the prevalence of cataract, pterygium, early and late age-related macular degeneration, diabetic retinopathy, and glaucoma was 40.2 ± 1.3%, 8.9 ± 0.5%, 5.1 ± 0.3%, 0.5 ± 0.1%, 13.4 ± 1.5%, and 2.1 ± 0.2%, respectively [8]. Although these progressive ophthalmic diseases are individually rare, they collectively account for a significant portion of global blindness. The proportion attributable to genetic causes remains unknown.

On the other hand, massively parallel sequencing (MPS), with its ability to test a large number of genes simultaneously in a cost-effective manner through massive parallelization, has significantly expedited the identification of underlying disease-causing mutations in patients with hereditary ophthalmic diseases. Several studies have established the diagnostic accuracy of MPS in hereditary ophthalmic diseases, yet their potential impact on treatment has been less explored [9,10,11,12]. In a general sense, three MPS methodologies aim to enhance diagnostics for heterogeneous diseases, such as targeted enrichment of specific gene sets (gene panels), whole exome sequencing (WES), and whole genome sequencing (WGS) [13]. Gene panel sequencing demonstrates exceptional efficacy in diagnosing genetically diverse hereditary ophthalmic diseases [14,15,16,17,18]. Targeted capture of known “disease genes” (referred to as “disease panels”), with its strong optimization of coverage on relevant targets, has demonstrated superiority over whole-exome sequencing in terms of read depth and on-target efficiency. By concurrently sequencing hundreds of genes potentially associated with diseases, gene panel sequencing offers a thorough examination of genetic profiles associated with the observed phenotypes [13]. For instance, Patel and colleagues developed the Oculome Panel Test, which comprises 429 known ophthalmic disease genes organized into five overlapping virtual subpanels. These subpanels cover genes associated with various conditions, including anterior segment developmental anomalies such as glaucoma (59 genes), microphthalmia–anophthalmia–coloboma (86 genes), congenital cataracts, and lens-associated conditions (70 genes), as well as retinal dystrophies (235 genes) and albinism (15 genes). Additionally, the panel includes extra genes linked to optic atrophy and complex strabismus (10 genes). Consequently, a wide spectrum of genetic conditions impacting eye development were genetically diagnosed, potentially replacing prolonged and expensive multidisciplinary assessments and enabling quicker targeted management [19]. Furthermore, its enhanced coverage, cost-effectiveness, and comparatively straightforward data interpretation have rendered gene panel sequencing more prevalent in standard clinical diagnostic practices compared to WES and WGS. Gene panel sequencing remains the preferred method for molecular genetic diagnostics of Mendelian disorders, primarily due to its capacity to accommodate more libraries per sequencing run while providing higher read depths compared to WES [20,21,22]. Additionally, it has been shown that panel-based genetic diagnostic testing for hereditary ophthalmic diseases is more sensitive for variant detection than WES [14,15]. Obtaining genetic diagnoses for patients with hereditary ophthalmic diseases is increasingly desirable for several reasons. First of all, it allows for the definition or confirmation of a clinical diagnosis, which may have prognostic value. Second, it facilitates precision in genetic counseling, aiding in disease management and family planning by determining the mode of inheritance. Third, it eliminates the need for costly, time-consuming, and potentially invasive diagnostic journeys that burden both families and the healthcare system. Consequently, the utilization of gene panel sequencing allows a greater number of patients to receive a genetic diagnosis for their clinical manifestations. As a good example, consider the application of gene panel sequencing for achieving a clinical diagnosis and assessing whether it influenced treatment decisions in Korean patients with hereditary ophthalmic diseases [18].

In this study, we investigated how to improve the yield of genetic diagnosis through additional gene panel sequencing in hereditary ophthalmic diseases.

## 2. Materials and Methods

### 2.1. Patient and DNA Extraction

Between 1 June 2018 and 30 August 2020, a cohort of 38 consecutive unrelated patients with hereditary ophthalmic diseases, with or without systemic conditions, who consented to gene panel sequencing, were included in this study. All patients underwent ophthalmologic examinations, which comprised slit-lamp examination, determination of the presence and type of nystagmus, identification of other systemic symptoms, fundus examination, and measurement of visual acuity. According to the manufacturer’s instructions, genomic DNA was isolated from peripheral blood samples using the QIAamp DNA mini kit (Qiagen, Hilden, Germany).

### 2.2. Library Preparation and Gene Panel Sequencing

**Figure 1:**
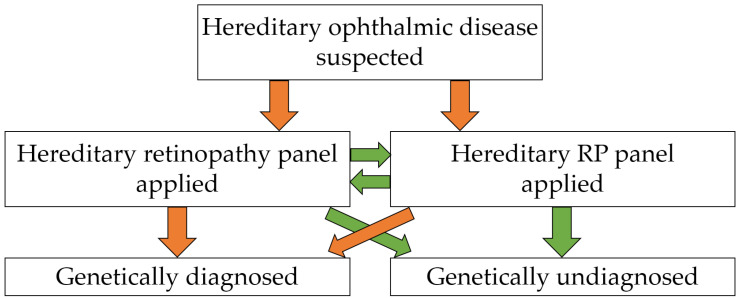
A flow diagram of genetic testing for the diagnosis of suspected hereditary ophthalmic disease was applied in this study. Orange arrows indicate positive results, while green arrows indicate negative results.

A gene panel sequencing consisting of a customized hereditary retinopathy panel or hereditary RP panel was prescribed and referred to a CAP-accredited clinical GC Genome laboratory (Yongin, Republic of Korea). If no significant mutations associated with hereditary retinopathy and RP were detected in either panel, additional gene panel sequencing was requested for research use, utilizing the remaining panel (Figure 1). Briefly, target enrichment was performed with custom-designed RNA oligonucleotide probes and a target enrichment kit (Celemics, Seoul, Republic of Korea). Pooled libraries were massively parallel sequenced using a MiSeqDX sequencer (Illumina, San Diego, CA, USA) and the MiSeqDx Reagent Kit v3 (Illumina, San Diego, CA, USA), which provides 150 bp × 2 paired-end reads. The gene list included in the hereditary retinopathy and RP panels is summarized in Supplementary Table S1.

### 2.3. Bioinformatic Analysis

Sequencing and bioinformatics analyses were conducted following the Genome Analysis Tool Kit best practice pipeline workflow (https://gatk.broadinstitute.org/hc/en-us; accessed on 7 January 2021), which encompassed processes such as base-calling, base alignment, variant calling, annotation, and quality control reporting. In short, sequences underwent alignment to the hg19 reference genome using BWA-aln. Single nucleotide variants and small insertions or deletions were identified and verified using GATK version 3.8.0 with Haplotypecaller, as well as VarScan version 2.4.0. The pathogenicity of missense variants was predicted using three in silico prediction algorithms, including SIFT (https://sift.bii.a-star.edu.sg/; accessed on 21 July 2021), PolyPhen2 (https://genetics.bwh.harvard.edu/pph2/; accessed on 21 July 2021), and MutationTester (https://www.mutationtaster.org/; accessed on 21 July 2021). Variant frequencies in the general population were assessed using the Genome Aggregation Database (gnomAD) (https://gnomad.broadinstitute.org/; accessed on 21 July 2021). Variant interpretation adhered to the five-tier classification system recommended by the American College of Medical Genetics and Genomics and the Association for Molecular Pathology [23]. Any variant deemed potentially likely pathogenic (LPV), pathogenic (PV), or of uncertain significance (VUS) was validated through visual examination of the BAM file using Integrated Genomics Viewer 2.3 software. In addition, small nucleotide substitution and insertion/deletion classified as LPV, PV, or VUS underwent further examination through Sanger sequencing. This process utilized a 3730xl Genetic Analyzer with the BigDye Terminator v3.1 Cycle Sequencing Kit (Applied Biosystems, Foster City, CA, USA). Subsequently, sequencing data were aligned to appropriate reference sequences and analyzed using Sequencher 5.3 software (Gene Codes Corp., Ann Arbor, MI, USA).

### 2.4. Segregation Analysis

The presence of the rare variant(s) in the proband was confirmed by bidirectional Sanger sequencing on a 3730xl DNA Analyzer (Applied Biosystems, Foster City, CA, USA). The origin of the rare variant(s) in the proband was determined by performing Sanger sequencing on the patient’s parents.

## 3. Results

Out of 38 patients with hereditary ophthalmic diseases, 23 (61%) were male, and 7 (18%) had family histories of similar phenotypes (patients ad5, fk2, yx9, gs6, so7, nm1, and mk4). All patients were of non-consanguineous parentage and Korean ethnicity. The cohort displayed phenotypic heterogeneity, with five with RP, four patients diagnosed with corneal dystrophy/cornea syndrome, three with macular dystrophy, two with congenital cataracts, and one with glaucoma. After additional gene panel sequencing, candidate variants consistent or inconsistent with clinical diagnosis were identified in 15 (39%) patients. A total of 16 heterozygous or homozygous variants were identified in 15 different genes associated with hereditary ophthalmic diseases (Table 1). Among these variants, the *PDE6B* variant is homozygous, and the *ZNF469* variant is compound heterozygous. The remaining variants are heterozygous. For the study’s purposes, patients were categorized into two groups: (1) presumptive genetic diagnosis, comprising cases with disease-associated PV or LPV whose phenotypes exactly matched their genotypes; and (2) unresolved cases, encompassing all other patients who did not have identified PV or LPV satisfying known Mendelian inheritance described in Online Mendelian Inheritance in Man (OMIM) (https://www.omim.org/; accessed on 3 September 2021). As a result, of 15 patients carrying any candidate variants, the clinical symptoms could be tentatively accounted for by genetic mutations in seven patients. However, in the remaining eight patients, given the in silico mutation predictive analysis, variant allele frequency in gnomAD, inheritance pattern, and genotype–phenotype correlation, fully elucidating the clinical manifestations with the identified rare variant was challenging.

### 3.1. Presumptively Genetically Diagnosed Hereditary Ophthalmic Diseases

Variations detected in the *ABCA4*, *CRYGD*, *MYOC*, *OCRL*, *PDE6B*, *RP1L1*, and *TGFBI* genes were capable of elucidating each patient’s disease. Examining each patient individually, heterozygous c.4297G>A/p.Val1433Ile of the *ABCA4* transmitted from the father was identified in a patient diagnosed with macular degeneration (Case ad5). This *ABCA4* variant was reported previously in a Stargardt patient carrying bialleic *ABCA4* variants, c.1302delA and c.4297G>A [24]. The heterozygous c.470G>A/p.Trp157Ter of the *CRYGD* inherited from the mother was identified in a patient diagnosed with early-onset cataract (Case fk2). This *CRYGD* variant was previously reported in congenital cataracts [25]. The heterozygous c.1021T>C/p. Ser341Pro of the *MYOC* transmitted from the father was identified in a patient diagnosed with primary open angle glaucoma (POAG) (Case yx9). This *MYOC* variant was reported previously in a Korean family with POAG [26]. The hemizygous c.2581G>A/p.Ala861Thr of the *OCRL* was identified in a patient suspected of Lowe syndrome (Case gs6). The asymptomatic mother of the proband was identified as an obligate heterozygote. Furthermore, his sister is an asymptomatic carrier. This *OCRL* variant was previously reported in a rare X-linked multi-systemic disorder, typically characterized by the triad of congenital cataract, cognitive and behavioral impairment, and proximal tubulopathy [27]. The homozygous c.1488del/p.Thr497ProfsTer78 of the *PDE6B* in a patient diagnosed with RP (Case so7). The asymptomatic parents of the proband were identified as obligate heterozygotes. This *PDE6B* was reported previously in Korean patients with *PDE6B*-associated RP [28]. The de novo heterozygous c.3971A>G/p.Glu1324Gly of the *RP1L1* was identified in a patient diagnosed with macular dystrophy (Case rq2). This *RP1L1* variant was previously reported in a patient with RP sine pigmento masquerading as moderate myopia [29]. The heterozygous c.371G>A/p.Arg124His of the *TGFBI* transmitted from the father was identified in a patient diagnosed with a very early stage of lattice dystrophy (Case nm1). This *TGFBI* variant is a hotspot mutation in the *TGFBI* gene, leading to the development of granular corneal dystrophy [30]. Candidate variants consistent with the clinical diagnosis in the presumptively genetically diagnosed seven patients with hereditary ophthalmic diseases are outlined in Table 2.

### 3.2. Hereditary Ophthalmic Diseases Left with an Undiagnosed Genetic Diagnosis

The identified variants alone in the *CDH23*, *CLRN1*, *LSS*, *OVOL2*, *PRDM5*, *TUB*, *TULP1*, and *ZNF469* genes were insufficient to genetically diagnose the symptoms of each patient. Particularly, the heterozygous c.9343A>G/p.Met3115Val of the *CDH23* was identified only in a patient diagnosed with RP (Case xf1). Segregation analysis was not available to the proband’s parents. This *CDH23* variant was previously reported in hearing loss using a comprehensive deafness proteome [31]. Furthermore, the *CDH23* gene is recognized as the gene responsible for autosomal recessive (AR) or digenic recessive Usher syndrome, specifically type 1D, in these individuals [32]. The heterozygous c.407G>A/p.Gly136Glu of *CLRN1* was identified only in a patient diagnosed with RP (Case as6). Segregation analysis was not available to the proband’s parents. This *CLRN1* variant was reported as a heterozygous variant with only one hit for the AR RP gene [16]. The heterozygous c.1120G>A/p.Asp374Asn of the *LSS* was identified only in a patient with a history of cataracts (Case ju6). Segregation analysis was not available to the proband’s parents. Biallelic mutations in *LSS* were first reported in families with congenital cataracts [33]. The heterozygous c.701A>T/p.Asn234Ile of *OVOL2* was identified in a patient diagnosed with macular dystrophy (Case wy5). Segregation analysis was not available to the proband’s parents. However, perturbed transcriptional regulation of *OVOL2* has been implicated as a major cause of dominant corneal endothelial dystrophies [34]. The heterozygous c.26G>A/p.Arg9Lys of *PRDM5* was identified only in a patient diagnosed with corneal dystrophy (Case jh3). Segregation analysis was not available to the proband’s parents. *PRDM5* mutations have been identified in families with Brittle Cornea Syndrome (BCS), an autosomal-recessive generalized connective tissue disorder [35]. The heterozygous c.1255C>T/p.Arg419Cys of the *TUB* was identified only in a patient diagnosed with RP (Case li1). Segregation analysis was not available to the proband’s parents. A recessive mutation in the *TUB* gene leads to obesity, deafness, and retinal degeneration [36]. The heterozygous c.349G>A/p.Glu117Lys of *TULP1* was identified only in a patient diagnosed with RP (Case pn3). Segregation analysis was not available to the proband’s parents. This *TULP1* variant was identified through WES in 168 Korean patients with hereditary retinal degeneration [20]. *TULP1* mutations could lead to a syndromic disorder, as evidenced by a recessive mutation in the *Tubby* gene in mice, which was associated not only with retinal degeneration but also with obesity, cochlear abnormalities, and diabetes [37]. The compound heterozygous c.9812C>T/p.Ala3271Val and c.10811C>T/p.Pro3604Leu of *ZNF469* was identified in a patient diagnosed with RP (Case mk4). The asymptomatic parents of the proband were identified as obligate heterozygotes. On family genetic testing, the two variants were found to be bi-allelic, but the clinical symptoms did not correspond to BCS. Heterozygous candidate variants consistent or inconsistent with the clinical diagnosis in eight patients with hereditary ophthalmic diseases, who remained undiagnosed genetically are delineated in Table 3.

## 4. Discussion

Nowadays, genetic diagnosis using MPS is widely employed, yet precision medicine remains largely inaccessible for most hereditary ophthalmic diseases. Gene panel sequencing involves isolating and analyzing targeted gene sets, offering a cost-effective alternative with reduced sequencing expenses. However, its success relies on the inclusion of disease-causing genes within the panel. A notable advantage is the minimized potential for incidental findings, coupled with the ability to achieve higher coverage at a lower cost compared to genome-wide approaches. While WES/WGS may be preferred, it hinges on ensuring that the lower coverage, in contrast to a gene panel, does not significantly diminish the diagnostic yield [13]. Our investigation revealed that the variant detection rate of targeted gene panel sequencing in hereditary ophthalmic diseases was approximately 39% (15 out of 38 cases). Among the 38 patients who underwent genetic testing, around 18% (7 out of 38) harbored candidate variants consistent with their clinical diagnosis, while 21% (8 out of 38) remained genetically undiagnosed. Fully elucidating the clinical manifestations with the identified rare variant proved challenging due to factors such as in silico mutation predictive analysis, variant allele frequency in gnomAD, inheritance pattern, and genotype–phenotype correlation.

In detail, five autosomal dominant (AD) ophthalmic diseases caused by variants in *ABCA4*, *CRYGD*, *MYOC*, *RPL1L1*, and *TGFBI*, one AR disease caused by the *PDE6B* variant, and one X-linked recessive disease caused by the *OCRL* variant were determined as genetic causes. In case ad5 with AD macular degeneration caused by *ABCA4*, reports indicate that *ABCA4* dominant heterozygous mutations may lead to age-related macular degeneration (AMD, MIM #153800) [38]. However, current hypotheses suggest that this condition could actually be a manifestation of very late-onset Stargardt disease, with mild and common hypomorphic alleles playing a role in pathogenicity [39]. *ABCA4* is responsible for causing over 95% of Stargardt disease 1 (STGD1, MIM #248200). Patients with variants in this gene may also exhibit different phenotypes, such as cone-rod dystrophy 3 (CRD3, MIM #604116), and RP 19 (MIM #601718). In case fk2 with AD early-onset cataract caused by *CRYGD*, crystallins represent the primary structural proteins within the human lens and are categorized into two families with distinct characteristics: the α-crystallins, which serve as chaperones, and the βγ-crystallins, which share the common structural unit Among these, γ-crystallins, the smallest and simplest members, are primarily localized in the nuclear region of the lens and possess two-domain structures. The solubility and stability of γD-crystallin are essential for maintaining lens transparency. Mutations in the *CRYGD* gene may compromise the solubility and stability of crystallin proteins, thereby reducing lens transparency and leading to congenital cataracts [40]. In case yx9 with AD POAG caused by *MYOC*, the findings from numerous empirical studies corroborate the assertion that a gain-of-function mechanism plays a role in the pathogenesis of myocilin-associated glaucoma [41]. Disease-causing myocilin variants have a propensity to aggregate and accumulate within the endoplasmic reticulum [42]. In cases where both wild-type (WT) and mutant myocilin coexist heterozygously within trabecular meshwork cells, the proteolytic processing and secretion of WT myocilin molecules are hindered. This impairment arises from interactions leading to the formation of hetero-oligomers between WT and mutant protein molecules [43]. In case rq2 with AD macular dystrophy caused by *RP1L1*, patients presenting with clinical symptoms of occult macular dystrophy (OCMD) caused by mutations in the *RP1L1* gene belong to the subgroup of occult macular dysfunction syndromes, also known as Miyake disease [44]. According to this classification, occult macular dysfunction syndrome can be subdivided into three categories: *RP1L1*-associated OCMD (Miyake disease), other hereditary OCMD caused by abnormalities in other genes, and non-hereditary occult macular dystrophy-like syndrome (progressive occult maculopathy). Characteristic clinical findings, including classical microstructural changes in spectral-domain optical coherence tomography images and an AD family history with reduced penetrance and variable expressivity, are important hallmarks of occult macular dysfunction syndromes associated with *RP1L1* [45]. In case nm1 with AD lattice dystrophy caused by *TGFBI*, the accumulation of transforming growth factor beta-induced protein (TGFBIp) is involved in the pathogenesis of TGFBI corneal dystrophies. The characteristic amyloid deposits observed in p.Arg124Cys and the non-amyloid (granular) deposits seen in p.Arg124His and p.Arg124Leu were linked to abnormal turnover and degradation of mutant TGFBIp [46]. According to published studies [47,48,49], the p.Arg124His mutation is the most frequently observed mutation in the Asian population. Previous Japanese studies have indicated that the p.Arg124His mutation is the most prevalent, constituting up to 72% of patients with corneal dystrophies [47]. In case so7 with AR RP caused by *PDE6B*, Korean RP patients caused by *PDE6B* variants exhibited symptoms earlier and were diagnosed earlier than patients with RP caused by other variants [28]. In the Korean RP cohort caused by *PDE6B* mutations, optical coherence tomography parameters revealed relatively frequent observations of epiretinal membranes and cystoid macular edema. This observation could be pertinent to *PDE6B* mutations because non-functional PDE6β subunits lead to an elevated intracellular level of cGMP, consequently resulting in increased Ca2+ influx due to decreased channel closure [28]. In case gs6 with X-linked recessive Lowe syndrome caused by *OCRL*, Lowe syndrome, a severe disorder, is characterized by congenital cataracts, mental disabilities, and hypotonia. In the common *OCRL* mutation, the c.2581G>A/p.Ala861Thr and c.2581G>C/p.Ala861Pro mutations result in the abolition of a 5′ splice site, leading to the skipping of exon 23 [50]. Understanding the consequences of exonic splicing mutations may hold potential therapeutic implications for patients with Lowe syndrome. Exon-skipping approaches, aimed at correcting mutations that disrupt normal pre-mRNA splicing, have been effectively evaluated in various rare diseases [51]. A successful exon-skipping strategy has been developed to restore significant levels of *OCRL* mRNA and protein in a Lowe syndrome patient with an intronic mutation. This mutation induces the incorporation of intronic sequences in the mRNA, ultimately leading to the loss of OCRL1 [52].

Our findings are consistent with previous research, suggesting that multiple genetic diagnoses can be identified through MPS [53]. It seems that many ophthalmologists lack awareness of which genetic profiles have actionable medical or surgical implications, and some may erroneously believe that genetic testing does not alter treatment approaches. Our study demonstrated that precise genetic diagnosis significantly impacts the understanding of molecular mechanisms, facilitating genotype-driven, tailored investigations. This, in turn, assists in averting secondary complications or associated medical conditions and reducing unnecessary treatments. It has been noted that the genetic diagnostic rate varies across different disease groups [54]. Typically, the detection rate of MPS is higher in hereditary ophthalmic diseases compared to other genetic conditions. In our study, the genetic diagnosis rate of hereditary ophthalmic diseases was not high, and various clinical phenotypes were genetically diagnosed. This observation contrasts with the findings of a previous study [19]. This difference may be attributed to the fact that clinicians specializing in specific ophthalmic diseases are not exclusively involved; rather, clinicians from various specialties treat patients with diverse ophthalmic disease conditions. Some individuals with rare hereditary ophthalmic diseases may receive an incorrect diagnosis or endure many years before reaching a definitive diagnosis [55,56]. Young children, in particular, are often not cooperative enough to undergo complete eye examinations or other diagnostic tests. Moreover, children with hereditary ophthalmic diseases may have underlying serious medical conditions that manifest with ocular symptoms. Consequently, they may undergo unnecessary brain imaging or numerous other investigations before arriving at a correct diagnosis [57]. For instance, Parekh and colleague [58] have established a clinical and referral workflow wherein each patient undergoes a coordinated evaluation by our multidisciplinary team, followed by discussions on diagnosis, prognosis, and genetic testing. The most frequently encountered referral diagnoses were congenital cataracts, optic neuropathy, and microphthalmia, with syndromic cases accounting for 52%. Within this patient cohort, a 76% uptake for genetic testing, with 33% of them receiving a diagnostic test result, was observed. These findings endorse the adoption of a personalized approach to genetic testing tailored to specific conditions.

Recent meta-analysis indicated that the diagnostic yield of MPS for hereditary retinopathy ranged from 61.3% in mixed hereditary retinal disease phenotypes to 58.2% in rod-cone dystrophies, 57.7% in macular and cone/cone-rod dystrophies, and 47.6% in familial exudative vitreoretinopathy [59]. Stone and his colleague [9] demonstrated that implementing a tier-based approach in genetic testing could enhance the genetic diagnostic yield. While we agree that a tier-based approach offers cost-effectiveness and higher diagnostic yields, it necessitates a high level of clinical expertise [60]. Furthermore, approximately 5% of patients with hereditary genetic diseases exhibited multi-locus genomic variations [53]. Even in cases where the clinical context suggests a single genetic etiology, targeted gene panel sequencing remains a valuable first-tier option because patients may harbor other ophthalmic conditions that are not evident during clinical examination. Panel-based genetic diagnostic testing for hereditary ophthalmic diseases is known for its high accuracy and reproducibility. Moreover, it is considered to be more sensitive for variant detection compared to exome sequencing [61]. Additionally, initial analysis focusing on genes well-established to be associated with a particular phenotype can enhance the positive predictive value and decrease the likelihood of false ascertainment [62]. However, regular updates to the target panel are necessary to incorporate new findings and advancements.

In several patients, we encountered challenges in determining the pathogenicity of variants. For instance, we identified a heterozygous c.407G>A/p.Gly136Glu of the *CLRN1* variant in a patient (as6) presenting with RP, a heterozygous c.1120G>A/p.Asp374Asn of the *LSS* variant in a patient (ju6) with cataract, and a heterozygous c.1255C>T/p.Arg419Cys of the *TUB* variant in a patient (li1) with RP. These missense variants were predicted to be deleterious or damaging by all three in silico tools, including SIFT, Polyphen2, and MutationTaster. However, bi-allelic variants in *CLRN1*, *LSS*, and *TUB* have been associated with AR Usher syndrome type 3 [63], congenital cataract [64], or RP, respecitvely [65]. Although these *CLRN1*, *LSS*, and *TUB* variants were rarely found in the population database, conducting functional analysis is essential to confirming their pathogenicity. With efforts to establish large population datasets like gnomAD, many variants previously considered pathogenic are now being reclassified as benign or likely benign [66]. On the other hand, in a patient (mk4) diagnosed with RP, in terms of genetic features, the presence of a compound heterozygous *ZNF469* variant was initially suspected as the cause of the clinical manifestation. However, the clinical features exhibited by the actual patient were completely different from what would typically be associated with this *ZNF469* gene.

Our study has several limitations. Firstly, the study design was retrospective, although our cases were collected consecutively by a single institution. Secondly, the majority of our patients belonged to a single Korean ethnicity, potentially limiting the generalizability of our findings to other populations. In addition, due to the limited number of patients, it was impossible to analyze whether the severity of clinical symptoms was influenced by gender or age of onset. In this study, there were no patients with diseases attributable to mutations in X chromosome-associated genes; however, for example, it was historically believed that female carriers of *RPGR* mutations had significantly milder, if any, symptoms compared to affected males with similar mutations. However, several reports indicate that female carriers can exhibit a spectrum of phenotypes, ranging from asymptomatic to severe retinal disease, similar to affected males. The presence of “affected” or partially manifesting female carriers without male-to-male transmission in a family lineage may lead to misinterpretation [67,68]. Thirdly, despite two gene panels (the hereditary retinopathy panel consisting of 193 genes and the hereditary RP panel consisting of 279 genes) applied consecutively, there is a possibility that newly discovered genes were not included in this panel-based sequencing approach. We evaluated medically or surgically actionable genes in ophthalmology based on the literature searches and GeneReviews (https://www.ncbi.nlm.nih.gov/books/NBK1116/; accessed on 13 April 2023). However, more comprehensive investigations or reviews of systematically curated databases are warranted to address these limitations. Comprehensive phenotyping, precise bioinformatics analysis, including known deep intronic variants, CNV detection, and cautious interpretations are crucial components of genetic diagnosis. Physicians should also be aware of the limitations of MPS, as it may not reliably detect variants in high-GC-rich regions, segmental duplications, or short tandem repeats. We concur that variants should be considered uncertain until proven otherwise.

## 5. Conclusions

In conclusion, our study highlights the utility of gene panel sequencing in achieving accurate diagnoses for hereditary ophthalmic diseases. Our approach enhances the diagnostic yield through additional gene panel sequencing. Performing thorough eye examinations can pose challenges, particularly for young patients, and some individuals may carry multiple genetic variations across unrelated genes, known as locus heterogeneity. Thus, gene panel sequencing can serve as a primary tool for the genetic diagnosis of hereditary ophthalmic diseases, even in cases where a single genetic cause is suspected. With a deeper comprehension of the genetic mechanisms underlying these diseases, it becomes feasible to offer more tailored treatments. The emergence of new gene therapy or pharmacogenetics studies holds promise for providing precision medicine to a broader patient population in the future, contingent upon the efficacy of these interventions being validated.

## Figures and Tables

**Table 1:** List of mutated genes registered in Online Mendelian Inheritance in Man (OMIM) associated with hereditary ophthalmic diseases.

Gene	Gene MIM	Clinical Phenotype	Phenotype MIM	Inheritance
*ABCA4*	*601691	{Macular degeneration, age-related, 2}	#153800	AD
		Cone-rod dystrophy 3	#604116	AR
*CDH23*	*605516	Usher syndrome, type 1D/F digenic	#601067	AR, DR
*CLRN1*	*606397	Retinitis pigmentosa 61	#614180	AR
*CRYGD*	*123690	Cataract 4, multiple types	#115700	AD
*LSS*	*600909	Cataract 44	#616509	AR
*MYOC*	*601652	Glaucoma 1A, primary open angle	#137750	AD
*OCRL*	*300535	Lowe syndrome	#309000	XLR
*OVOL2*	*616441	Corneal dystrophy, posterior polymorphous, 1	#122000	AD
*PDE6B*	*180072	Retinitis pigmentosa-40	#613801	AR
		Night blindness, congenital stationary, autosomal dominant 2	#163500	AD
*PRDM5*	*614161	Brittle cornea syndrome 2	#614170	AR
*RP1L1*	*608581	Occult macular dystrophy	#613587	AD
		Retinitis pigmentosa 88	#618826	AR
*TGFBI*	*601692	Corneal dystrophy, Avellino type	#607541	AD
*TUB*	*601197	Retinal dystrophy and obesity	#616188	AR
*TULP1*	*602280	Retinitis pigmentosa 14	#600132	AR
		Leber congenital amaurosis 15	#613843	AR
*ZNF469*	*612078	Brittle cornea syndrome 1	#229200	AR

Gene MIM and Phenotype MIM entries are denoted with the symbol * and #, respectively. MIM, Mendelian Inheritance in Man; AD, autosomal dominant; AR, autosomal recessive; DR, digenic recessive; XLR, x-linked recessive.

**Table 2:** Candidate variants consistent with clinical diagnosis in presumptively genetically diagnosed seven patients with hereditary ophthalmic diseases.

Case	S/A	FHx	Gene	Nucleotide ID	Base Change	Codon Change	dbSNP ID	ClinVar	S	PP2	MT	gnomAD
ad5	F/46	Pos	*ABCA4*	NM_000350.2	c.4297G>A	p.Val1433Ile	rs56357060	VUS	D	P	N	0.0017
fk2	F/33	Pos	*CRYGD*	NM_006891.3	c.470G>A	p.Trp157Ter	rs121909598	PV	na	na	na	0.0000
yx9	M/51	Pos	*MYOC*	NM_000261.1	c.1021T>C	p. Ser341Pro	rs1572210748	LPV	D	D	D	0.0000
gs6	M/19	Pos	*OCRL*	NM_000276.3	c.2581G>A	p.Ala861Thr	rs2124430527	PV	D	D	D	0.0000
so7	M/38	Pos	*PDE6B*	NM_000283.3	c.1488del	p.Thr497ProfsTer78	rs730880317	PV	na	na	na	0.0000
rq2	M/52	Neg	*RP1L1*	NM_178857.5	c.3971A>G	p.Glu1324Gly	rs4240659	VUS	T	B	P	0.0000
nm1	M/44	Pos	*TGFBI*	NM_000358.2	c.371G>A	p.Arg124His	rs121909211	PV	T	D	D	0.0000

S/A, sex/age; FHx, family history; Pos, positive; Neg, negative; S, SIFT_pred; PP2, Polyphen2_HDIV_pred; MT, MutationTaster_pred; gnomAD, gnomAD_v2.1.1, VUS, variant of uncertain significance; na, not available; PV, pathogenic variant; LPV, likely pathogenic variant; D, damaging; T, tolerant; P, pathogenic; B, benign; N, neutral.

**Table 3:** Candidate heterozygous variants consistent or inconsistent with clinical diagnosis in eight patients with hereditary ophthalmic diseases left with an undiagnosed genetic diagnosis.

Case	S/A	FHx	Gene	Nucleotide ID	Base Change	Codon Change	dbSNP ID	ClinVar	S	PP2	MT	gnomAD
xf1	F/49	Neg	*CDH23*	NM_022124.5	c.9343A>G	p.Met3115Val	rs772298163	na	D	B	D	0.0000
as6	F/51	Neg	*CLRN1*	NM_174878.2	c.407G>A	p.Gly136Glu	rs779258184	VUS	D	D	D	0.0001
ju6	M/34	Neg	*LSS*	NM_001001438.2	c.1120G>A	p.Asp374Asn	rs562778331	na	D	D	D	0.0000
wy5	M/51	Neg	*OVOL2*	NM_021220.3	c.701A>T	p.Asn234Ile	na	na	T	B	N	0.0000
jh3	M/47	Neg	*PRDM5*	NM_018699.3	c.26G>A	p.Arg9Lys	rs1037882347	na	D	B	D	0.0000
li1	M/44	Neg	*TUB*	NM_003320.4	c.1255C>T	p.Arg419Cys	rs1345174025	VUS	D	D	D	0.0000
pn3	M/36	Neg	*TULP1*	NM_003322.5	c.349G>A	p.Glu117Lys	rs527236117	LPV	T	B	D	0.0000
mk4	F/56	Pos	*ZNF469*	NM_001367624.2	c.9812C>T	p.Ala3271Val	rs547200758	VUS	T	B	N	0.0000
				NM_001367624.2	c.10811C>T	p.Pro3604Leu	rs957402222	VUS	T	P	N	0.0000

S/A, sex/age; FHx, family history; Neg, negative; Pos, positive; S, SIFT_pred; PP2, Polyphen2_HDIV_pred; MT, MutationTaster_pred; gnomAD, gnomAD_v2.1.1, VUS, variant of uncertain significance; na, not available; LPV, likely pathogenic variant; D, damaging; T, tolerant; P, pathogenic; B, benign; N, neutral.

## Data Availability

Data are contained within the article.

## Supplementary Materials

The following supporting information can be downloaded at: https://www.mdpi.com/article/10.3390/cimb46050300/s1. Supplementary Table S1. Gene list included in hereditary retinopathy and retinitis pigmentosa panel.

## References

[1] Stone E.M. (2007). Genetic testing for inherited eye disease. Arch. Ophthalmol..

[2] Kong L., Fry M., Al-Samarraie M., Gilbert C., Steinkuller P.G. (2012). An update on progress and the changing epidemiology of causes of childhood blindness worldwide. JAAPOS.

[3] Papadopoulos M., Cable N., Rahi J., Khaw P.T. (2007). The British Infantile and Childhood Glaucoma (BIG) Eye Study. Investig. Ophthalmol. Vis. Sci..

[4] Khordadpoor-Deilamani F., Akbari M.T., Karimipoor M., Javadi G. (2015). Sequence analysis of tyrosinase gene in ocular and oculocutaneous albinism patients: Introducing three novel mutations. Mol. Vis..

[5] Shah S.P., Taylor A.E., Sowden J.C., Ragge N.K., Russell-Eggitt I., Rahi J.S., Gilbert C.E. (2011). Anophthalmos, microphthalmos, and typical coloboma in the United Kingdom: A prospective study of incidence and risk. Investig. Ophthalmol. Vis. Sci..

[6] Méjécase C., Kozak I., Moosajee M. (2020). The genetic landscape of inherited eye disorders in 74 consecutive families from the United Arab Emirates. Am. J. Med. Genet. C Semin. Med. Genet..

[7] Hamblion E.L., Moore A.T., Rahi J.S. (2012). Incidence and patterns of detection and management of childhood-onset hereditary retinal disorders in the UK. Br. J. Ophthalmol..

[8] Yoon K.C., Mun G.H., Kim S.D., Kim S.H., Kim C.Y., Park K.H., Park Y.J., Baek S.H., Song S.J., Shin J.P. (2011). Prevalence of eye diseases in South Korea: Data from the Korea National Health and Nutrition Examination Survey 2008–2009. Korean J. Ophthalmol..

[9] Stone E.M., Andorf J.L., Whitmore S.S., DeLuca A.P., Giacalone J.C., Streb L.M., Braun T.A., Mullins R.F., Scheetz T.E., Sheffield V.C. (2017). Clinically Focused Molecular Investigation of 1000 Consecutive Families with Inherited Retinal Disease. Ophthalmology.

[10] Taylor R.L., Parry N.R.A., Barton S.J., Campbell C., Delaney C.M., Ellingford J.M., Hall G., Hardcastle C., Morarji J., Nichol E.J. (2017). Panel-Based Clinical Genetic Testing in 85 Children with Inherited Retinal Disease. Ophthalmology.

[11] Weisschuh N., Obermaier C.D., Battke F., Bernd A., Kuehlewein L., Nasser F., Zobor D., Zrenner E., Weber E., Wissinger B. (2020). Genetic architecture of inherited retinal degeneration in Germany: A large cohort study from a single diagnostic center over a 9-year period. Hum. Mutat..

[12] Del Pozo-Valero M., Riveiro-Alvarez R., Martin-Merida I., Blanco-Kelly F., Swafiri S., Lorda-Sanchez I., Trujillo-Tiebas M.J., Carreño E., Jimenez-Rolando B., Garcia-Sandoval B. (2022). Impact of Next Generation Sequencing in Unraveling the Genetics of 1036 Spanish Families with Inherited Macular Dystrophies. Investig. Ophthalmol. Vis. Sci..

[13] Sun Y., Ruivenkamp C.A., Hoffer M.J., Vrijenhoek T., Kriek M., van Asperen C.J., den Dunnen J.T., Santen G.W. (2015). Next-generation diagnostics: Gene panel, exome, or whole genome?. Hum. Mutat..

[14] Jespersgaard C., Fang M., Bertelsen M., Dang X., Jensen H., Chen Y., Bech N., Dai L., Rosenberg T., Zhang J. (2019). Molecular genetic analysis using targeted NGS analysis of 677 individuals with retinal dystrophy. Sci. Rep..

[15] Wang P., Li S., Sun W., Xiao X., Jia X., Liu M., Xu L., Long Y., Zhang Q. (2019). An Ophthalmic Targeted Exome Sequencing Panel as a Powerful Tool to Identify Causative Mutations in Patients Suspected of Hereditary Eye Diseases. Transl. Vis. Sci. Technol..

[16] Dan H., Huang X., Xing Y., Shen Y. (2020). Application of targeted panel sequencing and whole exome sequencing for 76 Chinese families with retinitis pigmentosa. Mol. Genet. Genom. Med..

[17] Kim Y.J., Kim Y.N., Yoon Y.H., Seo E.J., Seo G.H., Keum C., Lee B.H., Lee J.Y. (2021). Diverse Genetic Landscape of Suspected Retinitis Pigmentosa in a Large Korean Cohort. Genes.

[18] Moon D., Park H.W., Surl D., Won D., Lee S.T., Shin S., Choi J.R., Han J. (2021). Precision Medicine through Next-Generation Sequencing in Inherited Eye Diseases in a Korean Cohort. Genes.

[19] Patel A., Hayward J.D., Tailor V., Nyanhete R., Ahlfors H., Gabriel C., Jannini T.B., Abbou-Rayyah Y., Henderson R., Nischal K.K. (2019). The Oculome Panel Test: Next-Generation Sequencing to Diagnose a Diverse Range of Genetic Developmental Eye Disorders. Ophthalmology.

[20] Ma D.J., Lee H.S., Kim K., Choi S., Jang I., Cho S.H., Yoon C.K., Lee E.K., Yu H.G. (2021). Whole-exome sequencing in 168 Korean patients with inherited retinal degeneration. BMC Med. Genom..

[21] Suga A., Yoshitake K., Minematsu N., Tsunoda K., Fujinami K., Miyake Y., Kuniyoshi K., Hayashi T., Mizobuchi K., Ueno S. (2022). Genetic characterization of 1210 Japanese pedigrees with inherited retinal diseases by whole-exome sequencing. Hum Mutat..

[22] Yang E., Yu J., Liu X., Chu H., Li L. (2023). Familial Whole Exome Sequencing Study of 30 Families with Early-Onset High Myopia. Investig. Ophthalmol. Vis. Sci..

[23] Richards S., Aziz N., Bale S., Bick D., Das S., Gastier-Foster J., Grody W.W., Hegde M., Lyon E., Spector E. (2015). Standards and guidelines for the interpretation of sequence variants: A joint consensus recommendation of the American College of Medical Genetics and Genomics and the Association for Molecular Pathology. Genet. Med..

[24] Buhler V.M.M., Berger L., Schaller A., Zinkernagel M.S., Wolf S., Escher P. (2021). Absence of Genotype/Phenotype Correlations Requires Molecular Diagnostic to Ascertain Stargardt and Stargardt-like Swiss Patients. Genes.

[25] Santhiya S.T., Shyam Manohar M., Rawlley D., Vijayalakshmi P., Namperumalsamy P., Gopinath P.M., Löster J., Graw J. (2002). Novel mutations in the gamma-crystallin genes cause autosomal dominant congenital cataracts. J. Med. Genet..

[26] Moon S., Kim N., Lee J. (2020). Clinical and genetic analysis of Ser341Pro MYOC variant in a Korean family with primary open angle glaucoma. Int. J. Ophthalmol..

[27] Recker F., Zaniew M., Böckenhauer D., Miglietti N., Bökenkamp A., Moczulska A., Rogowska-Kalisz A., Laube G., Said-Conti V., Kasap-Demir B. (2015). Characterization of 28 novel patients expands the mutational and phenotypic spectrum of Lowe syndrome. Pediatr. Nephrol..

[28] Kim Y.N., Song J.S., Oh S.H., Kim Y.J., Yoon Y.H., Seo E.J., Seol C.A., Lee S.M., Choi J.M., Seo G.H. (2020). Clinical characteristics and disease progression of retinitis pigmentosa associated with PDE6B mutations in Korean patients. Sci. Rep..

[29] Lu Y., Sun X. (2021). Retinitis pigmentosa sine pigmento masqueraded as myopia: A case report (CARE). Medicine.

[30] Munier F.L., Korvatska E., Djemaï A., Le Paslier D., Zografos L., Pescia G., Schorderet D.F. (1997). Kerato-epithelin mutations in four 5q31-linked corneal dystrophies. Nat. Genet..

[31] Tollefson M.R., Gogal R.A., Weaver A.M., Schaefer A.M., Marini R.J., Azaiez H., Kolbe D.L., Wang D., Weaver A.E., Casavant T.L. (2023). Assessing variants of uncertain significance implicated in hearing loss using a comprehensive deafness proteome. Hum. Genet..

[32] Bolz H., von Brederlow B., Ramírez A., Bryda E.C., Kutsche K., Nothwang H.G., Seeliger M., del C.S.C.M., Vila M.C., Molina O.P. (2001). Mutation of CDH23, encoding a new member of the cadherin gene family, causes Usher syndrome type 1D. Nat. Genet..

[33] Zhao L., Chen X.J., Zhu J., Xi Y.B., Yang X., Hu L.D., Ouyang H., Patel S.H., Jin X., Lin D. (2015). Lanosterol reverses protein aggregation in cataracts. Nature.

[34] Davidson A.E., Liskova P., Evans C.J., Dudakova L., Nosková L., Pontikos N., Hartmannová H., Hodaňová K., Stránecký V., Kozmík Z. (2016). Autosomal-Dominant Corneal Endothelial Dystrophies CHED1 and PPCD1 Are Allelic Disorders Caused by Non-coding Mutations in the Promoter of OVOL2. Am. J. Hum. Genet..

[35] Burkitt Wright E.M.M., Spencer H.L., Daly S.B., Manson F.D.C., Zeef L.A.H., Urquhart J., Zoppi N., Bonshek R., Tosounidis I., Mohan M. (2011). Mutations in PRDM5 in brittle cornea syndrome identify a pathway regulating extracellular matrix development and maintenance. Am. J. Hum. Genet..

[36] Hagstrom S.A., North M.A., Nishina P.L., Berson E.L., Dryja T.P. (1998). Recessive mutations in the gene encoding the tubby-like protein TULP1 in patients with retinitis pigmentosa. Nat. Genet..

[37] Jacobson S.G., Cideciyan A.V., Huang W.C., Sumaroka A., Roman A.J., Schwartz S.B., Luo X., Sheplock R., Dauber J.M., Swider M. (2014). TULP1 mutations causing early-onset retinal degeneration: Preserved but insensitive macular cones. Investig. Ophthalmol. Vis. Sci..

[38] Allikmets R., Shroyer N.F., Singh N., Seddon J.M., Lewis R.A., Bernstein P.S., Peiffer A., Zabriskie N.A., Li Y., Hutchinson A. (1997). Mutation of the Stargardt disease gene (ABCR) in age-related macular degeneration. Science.

[39] Zernant J., Lee W., Collison F.T., Fishman G.A., Sergeev Y.V., Schuerch K., Sparrow J.R., Tsang S.H., Allikmets R. (2017). Frequent hypomorphic alleles account for a significant fraction of ABCA4 disease and distinguish it from age-related macular degeneration. J. Med. Genet..

[40] Wang K.J., Wang J.X., Wang J.D., Li M., Zhang J.S., Mao Y.Y., Wan X.H. (2023). Congenital coralliform cataract is the predominant consequence of a recurrent mutation in the CRYGD gene. Orphanet J. Rare Dis..

[41] Sharma R., Grover A. (2021). Myocilin-associated Glaucoma: A Historical Perspective and Recent Research Progress. Mol. Vis..

[42] Joe M.K., Sohn S., Hur W., Moon Y., Choi Y.R., Kee C. (2003). Accumulation of mutant myocilins in ER leads to ER stress and potential cytotoxicity in human trabecular meshwork cells. Biochem. Biophys. Res. Commun..

[43] Gobeil S., Rodrigue M.A., Moisan S., Nguyen T.D., Polansky J.R., Morissette J., Raymond V. (2004). Intracellular sequestration of hetero-oligomers formed by wild-type and glaucoma-causing myocilin mutants. Investig. Ophthalmol. Vis. Sci..

[44] Fujinami K., Kameya S., Kikuchi S., Ueno S., Kondo M., Hayashi T., Shinoda K., Machida S., Kuniyoshi K., Kawamura Y. (2016). Novel RP1L1 Variants and Genotype-Photoreceptor Microstructural Phenotype Associations in Cohort of Japanese Patients With Occult Macular Dystrophy. Investig. Ophthalmol. Vis. Sci..

[45] Zobor D., Zobor G., Hipp S., Baumann B., Weisschuh N., Biskup S., Sliesoraityte I., Zrenner E., Kohl S. (2018). Phenotype Variations Caused by Mutations in the RP1L1 Gene in a Large Mainly German Cohort. Investig. Ophthalmol. Vis. Sci..

[46] Korvatska E., Henry H., Mashima Y., Yamada M., Bachmann C., Munier F.L., Schorderet D.F. (2000). Amyloid and non-amyloid forms of 5q31-linked corneal dystrophy resulting from kerato-epithelin mutations at Arg-124 are associated with abnormal turnover of the protein. J. Biol. Chem..

[47] Fujiki K., Nakayasu K., Kanai A. (2001). Corneal dystrophies in Japan. J. Hum. Genet..

[48] Song J.S., Lim D.H., Chung E.S., Chung T.Y., Ki C.S. (2015). Mutation Analysis of the TGFBI Gene in Consecutive Korean Patients With Corneal Dystrophies. Ann. Lab. Med..

[49] Li W., Qu N., Li J.K., Li Y.X., Han D.M., Chen Y.X., Tian L., Shao K., Yang W., Wang Z.S. (2021). Evaluation of the Genetic Variation Spectrum Related to Corneal Dystrophy in a Large Cohort. Front. Cell Dev. Biol..

[50] Suarez-Artiles L., Perdomo-Ramirez A., Ramos-Trujillo E., Claverie-Martin F. (2018). Splicing Analysis of Exonic OCRL Mutations Causing Lowe Syndrome or Dent-2 Disease. Genes.

[51] Veltrop M., Aartsma-Rus A. (2014). Antisense-mediated exon skipping: Taking advantage of a trick from Mother Nature to treat rare genetic diseases. Exp. Cell Res..

[52] Rendu J., Montjean R., Coutton C., Suri M., Chicanne G., Petiot A., Brocard J., Grunwald D., Pietri Rouxel F., Payrastre B. (2017). Functional Characterization and Rescue of a Deep Intronic Mutation in OCRL Gene Responsible for Lowe Syndrome. Hum. Mutat..

[53] Posey J.E., Harel T., Liu P., Rosenfeld J.A., James R.A., Coban Akdemir Z.H., Walkiewicz M., Bi W., Xiao R., Ding Y. (2017). Resolution of Disease Phenotypes Resulting from Multilocus Genomic Variation. N. Engl. J. Med..

[54] Smedley D., Smith K.R., Martin A., Thomas E.A., McDonagh E.M., Cipriani V., Ellingford J.M., Arno G., Tucci A., Vandrovcova J. (2021). 100,000 Genomes Pilot on Rare-Disease Diagnosis in Health Care—Preliminary Report. N. Engl. J. Med..

[55] Men C.J., Bujakowska K.M., Comander J., Place E., Bedoukian E.C., Zhu X., Leroy B.P., Fulton A.B., Pierce E.A. (2017). The importance of genetic testing as demonstrated by two cases of CACNA1F-associated retinal generation misdiagnosed as LCA. Mol. Vis..

[56] Miraldi Utz V., Pfeifer W., Longmuir S.Q., Olson R.J., Wang K., Drack A.V. (2018). Presentation of TRPM1-Associated Congenital Stationary Night Blindness in Children. JAMA Ophthalmol..

[57] Rim J.H., Lee S.T., Gee H.Y., Lee B.J., Choi J.R., Park H.W., Han S.H., Han J. (2017). Accuracy of Next-Generation Sequencing for Molecular Diagnosis in Patients with Infantile Nystagmus Syndrome. JAMA Ophthalmol..

[58] Parekh B., Beil A., Blevins B., Jacobson A., Williams P., Innis J.W., Barone Pritchard A., Prasov L. (2023). Design and Outcomes of a Novel Multidisciplinary Ophthalmic Genetics Clinic. Genes.

[59] Britten-Jones A.C., Gocuk S.A., Goh K.L., Huq A., Edwards T.L., Ayton L.N. (2023). The Diagnostic Yield of Next Generation Sequencing in Inherited Retinal Diseases: A Systematic Review and Meta-analysis. Am. J. Ophthalmol..

[60] Moore A.T. (2017). Genetic Testing for Inherited Retinal Disease. Ophthalmology.

[61] Consugar M.B., Navarro-Gomez D., Place E.M., Bujakowska K.M., Sousa M.E., Fonseca-Kelly Z.D., Taub D.G., Janessian M., Wang D.Y., Au E.D. (2015). Panel-based genetic diagnostic testing for inherited eye diseases is highly accurate and reproducible, and more sensitive for variant detection, than exome sequencing. Genet. Med..

[62] Weck K.E. (2018). Interpretation of genomic sequencing: Variants should be considered uncertain until proven guilty. Genet. Med..

[63] Herrera W., Aleman T.S., Cideciyan A.V., Roman A.J., Banin E., Ben-Yosef T., Gardner L.M., Sumaroka A., Windsor E.A., Schwartz S.B. (2008). Retinal disease in Usher syndrome III caused by mutations in the clarin-1 gene. Investig. Ophthalmol. Vis. Sci..

[64] Zhao M., Mei T., Shang B., Zou B., Lian Q., Xu W., Wu K., Lai Y., Liu C., Wei L. (2021). Defect of LSS Disrupts Lens Development in Cataractogenesis. Front. Cell Dev. Biol..

[65] North M.A., Naggert J.K., Yan Y., Noben-Trauth K., Nishina P.M. (1997). Molecular characterization of TUB, TULP1, and TULP2, members of the novel tubby gene family and their possible relation to ocular diseases. Proc. Natl. Acad. Sci. USA.

[66] Whiffin N., Minikel E., Walsh R., O’Donnell-Luria A.H., Karczewski K., Ing A.Y., Barton P.J.R., Funke B., Cook S.A., MacArthur D. (2017). Using high-resolution variant frequencies to empower clinical genome interpretation. Genet. Med..

[67] Al-Maskari A., O’Grady A., Pal B., McKibbin M. (2009). Phenotypic progression in X-linked retinitis pigmentosa secondary to a novel mutation in the RPGR gene. Eye.

[68] Churchill J.D., Bowne S.J., Sullivan L.S., Lewis R.A., Wheaton D.K., Birch D.G., Branham K.E., Heckenlively J.R., Daiger S.P. (2013). Mutations in the X-linked retinitis pigmentosa genes RPGR and RP2 found in 8.5% of families with a provisional diagnosis of autosomal dominant retinitis pigmentosa. Investig. Ophthalmol. Vis. Sci..

