# Supplementary material for: Improving the Yield of Genetic Diagnosis through Additional Genetic Panel Testing in Hereditary Ophthalmic Diseases
Source: Curr Issues Mol Biol. 2024 May 20;46(5):5010–22. doi: 10.3390/cimb46050300 (PMC11119902; doi:10.3390/cimb46050300)
Supplement: Supplementary file 1 [file cimb-46-00300-s001.zip › cimb-2976907-supplementary.pdf]

Supplementary Table S1. Gene list included in hereditary retinopathy and retinitis pigmentosa panel

Hereditary retinopathy panel (n=193)
ABCB6, ACTB, ACTG1, ADAMTS10, ADAMTS17, ADAMTS18, ADAMTSL4, ALDH1A3, ATOH7, B3GALNT2, B3GLCT, BCOR, BEST1, BMP4, C12orf57, CBS, CC2D2A, CEP290, CHD7, CHRDL1, CHST6, CLDN19, COL18A1, COL4A1, CPAMD8, CRPPA, CRYAA, CRYAB, CRYGC, CYP1B1, DDX58, DOCK6, ESCO2, FAT1, FBN1, FKTN, FOXC1, FOXD3, FOXE3, FRAS1, FREM1, FREM2, GDF6, GJA1, GJA8, GRIP1, HCCS, HMX1, INPP5E, KIAA1109, KMT2D, LAMB2, LRP2, LRP5, LSS, LTBP2, MAB21L2, MAF, MAPRE2, MFRP, MITF, MYOC, MYRF, NAA10, NDP, NHS, OPA1, OTX2, OVOL2, PAX2, PAX6, PIGL, PITX2, PITX3, POMGNT1, POMT1, POMT2, PORCN, PRDM5, PRR12, PRSS56, PTCH1, PUF60, PXDN, RAB18, RAB3GAP1, RAB3GAP2, RARB, RAX, RBP4, RERE, RIPK4, RPGRIP1L, SALL1, SALL4, SBF2, SH3PXD2B, SHH, SIX6, SLC38A8, SLC4A4, SMCHD1, SMO, SMOC1, SOX2, SRD5A3, STRA6, TBC1D20, TENM3, TFAP2A, TGFBI, TMEM216, TMEM237, TMEM67, TMEM98, TUBGCP4, VSX2, YAP1, ZEB2, AIPL1, ALX1, B3GALT1, BMP7, BMPR1A, CAPN15, CDH2, CDK5RAP2, CDON, CENPF, CNNM4, COL2A1, COL6A3, COX7B, CRB1, CREBBP, CRIM1, CRYBA4, CRYBB1, CRYBB2, CRYGD, DAG1, DYRK1A, EFTUD2, ERCC1, FANCL, FKRP, FZD4, FZD5, GDF3, GLI2, HMGB3, IFIH1, IGBP1, IPO13, KDM6A, KERA, KIAA0586, KIF11, LMX1B, MIR204, NMNAT1, NUP188, OCRL, OFD1, OLFM2, PDE6D, PLK4, POMGNT2, PQBP1, RHOA, SALL2, SCLT1, SIX3, SLC16A12, SLC25A24, SLC2A1, SMAD4, SMG9, TBC1D32, TCOF1, TEK, TMX3, TOGARAM1, TUBB, VAX1, VPS35L, VSX1, WDR37, WNT2B, WRAP73, ZEB1, ZIC2, ZNF469
Hereditary retinitis pigmentosa panel (n=279)
ABCA4, ABHD12, ACO2, ADAM9, ADAMTS18, ADGRV1, AGBL5, AHI1, AIPL1, AIRE, ALMS1, ARHGEF18, ARL2BP, ARL6, ATF6, ATOH7, BBS1, BBS10, BBS12, BBS2, BBS4, BBS5, BBS7, BBS9, BEST1, C1QTNF5, CABP4, CACNA1F, CACNA2D4, CAPN5, CC2D2A, CDH23, CDH3, CDHR1, CEP164, CEP290, CEP78, CERKL, CFAP410, CFAP418, CFH, CHM, CIB2, CLN3, CLN5, CLN6, CLN8, CLRN1, CNGA1, CNGA3, CNGB1, CNGB3, CNNM4, COL18A1, COL4A1, CRB1, CRX, CSPP1, CTNNB1, CTSD, CWC27, CYP4V2, DHDDS, EFEMP1, ELOVL4, ERCC6, ERCC8, EYS, FAM161A, FLVCR1, FZD4, GNAT1, GNAT2, GNPTG, GPR143, GPR179, GRK1, GRM6, GUCA1A, GUCA1B, GUCY2D, HARS1, HCCS, HGSNAT, HMX1, IDH3A, IDH3B, IFT140, IKBKG, IMPDH1, IMPG1, IMPG2, INPP5E, IQCB1, KCNJ13, KCNV2, KIAA1549, KIF11, KIZ, KLHL7, LCA5, LRAT, LRIT3, LRP2, LRP5, LZTFL1, MAK, MERTK, MFRP, MFSD8, MKKS, MKS1, MYO7A, NDP, NMNAT1, NPHP1, NPHP3, NPHP4, NR2E3, NRL, NYX, OAT, OFD1, OPN1LW, OPN1MW, OTX2, PANK2, PCARE, PCDH15, PCYT1A, PDE6A, PDE6B, PDE6C, PDE6G, PEX1, PEX2, PEX7, PHYH, PLA2G5, POC1B, PPT1, PRCD, PROM1, PRPF3, PRPF4, PRPF6, PRPF8, PRPS1, RAB28, RAX2, RBP3, RBP4, RCBTB1, RD3, RDH12, RDH5, REEP6, RGS9, RLBP1, RP1L1, RP9, RPE65, RPGRIP1, RPGRIP1L, RS1, SAG, SCAPER, SDCCAG8, SLC24A1, SLC38A8, SNRNP200, SPATA7, SRD5A3, TIMM8A, TIMP3, TMEM237, TOPORS, TPP1, TRIM32, TRPM1, TSPAN12, TTC8, TTLL5, TUB, TULP1, USH1C, USH1G, VCAN, VPS13B, WDPCP, WDR19, WHRN, ZNF408, ZNF423, ABCC6, ACBD5, ADIPOR1, AFG3L2, AHR, ALPK1, AMACR, ARL13B, ARL3, ARSG, ASRGL1, ATXN7, CA4, CCT2, CEP19, CEP250, CLCC1, CLUAP1, CTC1, CTNNA1, CYP2R1, DHX38, DMD, DRAM2, ELOVL1, ESPN, EXOSC2, GDF6, GNB3, GRN, HK1, IFT172, IFT27, IFT74, IFT81, JAG1, KIF3B, LAMA1, LIG3, MAPKAPK3, MIR204, MMACHC, MSTO1, MTRFR, MTTP, MVK, NBAS, NEUROD1, OPN1SW, P3H2, PAX2, PDE6H, PEX6, PGK1, PLK4, PNPLA6, POC5, POMGNT1, PRDM13, RDH11, RGR, RIMS2, ROM1, RTN4IP1, SAMD11, SEMA4A, SLC25A46, SLC37A3, SLC6A6, SPP2, SSBP1, TINF2, TLCD3B, TMEM216, TMEM231, TRAF3IP1, TREX1, TRNT1, TTPA, TUBB4B, TUBGCP4, TUBGCP6, UNC119, USP45, ZFYVE26
